# Coordinating a Postgraduate Orthopaedic Exam During the COVID-19 Pandemic

**DOI:** 10.5704/MOJ.2103.003

**Published:** 2021-03

**Authors:** NH Mohamed-Haflah, S Abdullah, R Abdul-Rani

**Affiliations:** Department of Orthopaedics and Traumatology, Universiti Kebangsaan Malaysia, Kuala Lumpur, Malaysia

**Keywords:** COVID-19, exam, national board, pandemic, standard operating procedure

## Abstract

The Coronavirus (COVID-19) pandemic and institution of the Movement Control Order (MCO) had resulted in the cancellation of a major orthopaedic exam in April 2020. The exam is known as the Malaysian Orthopaedic Specialist Committee (OSC) Part I Examinations. It is similar to the British Royal Colleges of Surgeons Membership (MRCS) exams and held twice annually in April and October. There are up to 200 candidates involved. With implementation of new guidelines and standard operating procedures (SOP), the OSC Part I exam was successfully held by Universiti Kebangsaan Malaysia (UKM) from 5th-9th October 2020. Here we highlight the challenges we faced whilst coordinating a major exam at a national level during the COVID-19 pandemic with recommendations for future exams.

## Introduction

The Orthopaedic Master Programme in Malaysia is a four-year programme and is one of the requirements for surgeons to practice orthopaedic surgery. Over the course of these four years, students will cover eight subspecialties which are trauma, arthroplasty, sports, foot and ankle, orthopaedic oncology, hand and microsurgery and spine and paediatrics. In addition, they are required to pass the Orthopaedic Specialist Committee (OSC) Part 1 and Part II Examinations. The OSC Part I exam covers anatomy, physiology, pathology and basic surgical principles. It is similar to the British Royal Colleges of Surgeons Membership (MRCS) exams but emphasising more on orthopaedic surgery. Held twice annually in April and October it is hosted by one of the university hospitals. The exam consists of three parts which are theory, objective structured clinical examination (OSCE) and viva. Passing all three components are mandatory to pass the entire exam.

Coronavirus (COVID-19) has caused major disruption to worldwide socioeconomics^1,2^. In orthopaedic and trauma surgery, adaptation was required and changes had to be implemented to allow return to a ‘new normal’ way of life^3-5^. The first confirmed case of coronavirus (COVID-19) infection in Malaysia was on 25th January 2020 when a group of travellers from China entered Malaysia^6^. The country instituted the Movement Control Order (MCO) on March 18th which banned non-essential travel and large gatherings including examinations. Consequently, the OSC Part I exam as scheduled for April 2020 was cancelled. This and also cancellation of the OSC Part II exam required adjustment to be made to the entire Orthopaedic Master Programme with respect to academic year progression and also graduation of new specialists. With implementation of various public health measures, the curve flattened and the country began to adjust back to a ‘new normal’ way of life. Gatherings with a maximum of 200 people in an adequate space to allow social distancing of minimum one metre were allowed. At this point our committee decided to hold the scheduled October exams albeit with a sense of apprehension. The exam hosted by Universiti Kebangsaan Malaysia (UKM) was to be held in UKM Medical Centre (UKMMC) from 5th to 9th October 2020.

## Exam Planning and Preparation

### Venue

**Fig. 1:**
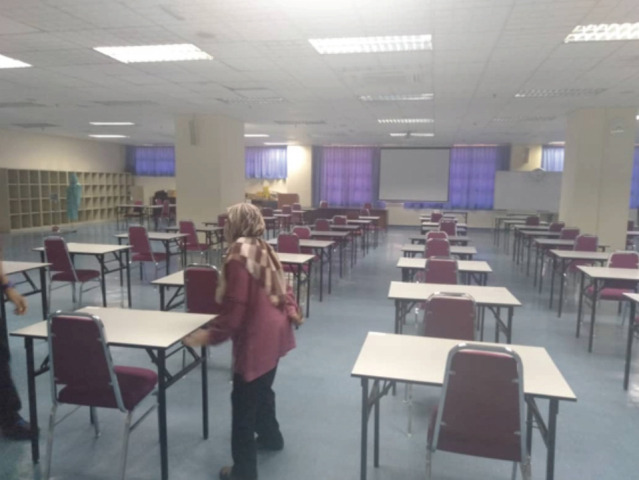
Setting up for the theory paper.

Adherence to the standard operating procedures (SOP) of ensuring social distancing of a minimum of one metre apart, adequate space was required for all components of the exam. In the past up to 200 candidates had registered for the exam. Thus, we had to physically measure the exam hall to calculate the number of candidates that we could fit in for the theory section. After assessment, we took 140 as a cut-off point (Fig. 1). A larger than normal area was also required for registration since all candidates had to be one metre apart during the registration.

According to statistics, the passing rate of the theory section is approximately 50-60%. Only those that pass the theory section will proceed to the OSCE and Viva section which is held in the examination ward. There are two examination wards in UKMMC designated as centre 1 and centre 2 which are connected with a double door. Each examination ward has 10 rooms, 5 six-bedded cubicles and 1 conference room. OSCE consists of eight stations which tests students on basic orthopaedic procedures and skills and is usually held in a small room. Since only one examiner is required, along with the candidate, the size of the room would ensure the adherence of the one metre rule. To minimise person to person contact, stations requiring standardised patients were replaced with other medium. On these occasions, photos were used to simulate clinical conditions.

**Fig. 2:**
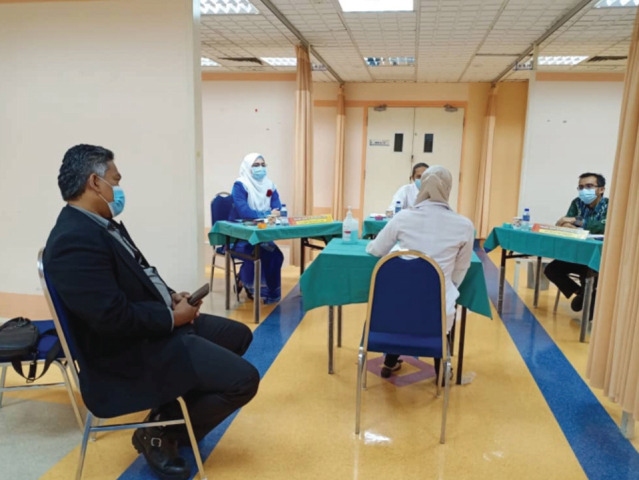
Viva sessions conducted in ward cubicles.

**Fig. 3:**
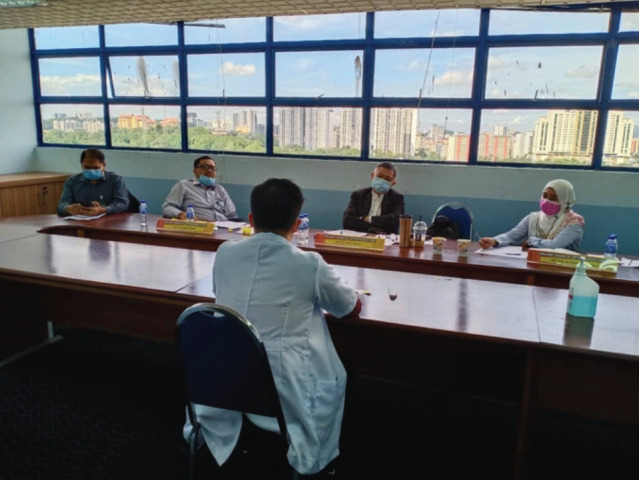
Viva sessions conducted in a large room.

**Fig. 4:**
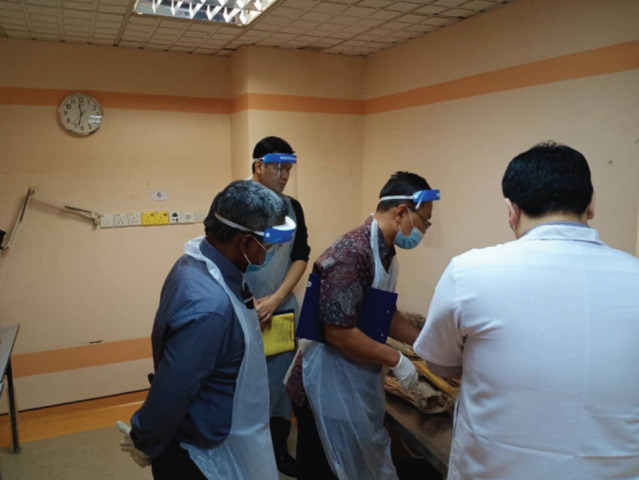
Protective eyewear, mask, apron and gloves were required for anatomy vivas.


The viva consists of three sections which are clinical anatomy, clinical physiology and principles of orthopaedic surgery. Three examiners are required for each section; in addition there will occasionally be an observer in these sessions. Vivas are normally conducted in the same room as the OSCE. However, in order to comply with the SOP this would not be possible. Instead the viva was relocated to the examination ward cubicle requiring the use of both examination centres (Fig. 2 and 3). Nevertheless, it would be difficult to comply with social distancing for the Anatomy station. Since cadaveric specimens were used, examiners were expected to stand closer to the candidates. In order to ensure safety, face shields, aprons and gloves would have to be provided (Fig. 4).

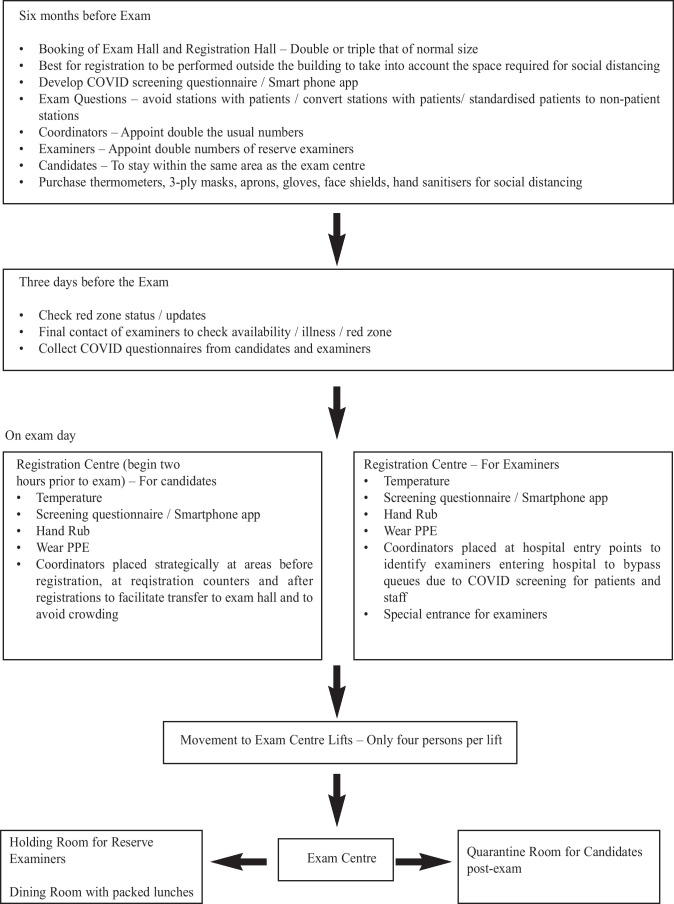


After completion of the exam, candidates are required to be quarantined in two or three rooms depending on the numbers. For this exam we used the conference room in our department which was situated one level below the exam hall. In short, the venue space required to conduct the exam was approximately double compared to normal circumstances.

### Equipment

The standard measures to ensure safety and reduction of virus transmission was also implemented. We purchased a mass thermometer scan, 3-ply masks, alcohol rubs, aprons, gloves and face shields. We were also required to register for a QR code for scanning.

And then everything went haywire^7^…

## Implementation of New Guidelines and SOP

Ten days prior to the exam, Sabah, a state in East Malaysia had a surge of COVID-19 cases ringing alarm bells^7^. The government stipulated that Sabahans entering Peninsular Malaysia (exam venue) had to be quarantined for 14 days. This made it impossible for Sabahan candidates and examiners to attend the exams. In addition to the 17 candidates who were unable to take the exams, our external examiner also had to be excused from attending the exam. In light of this ruling from our government, the Deans’ Council came out with a new SOP to address the above issues. All candidates were required to fill in a health questionnaire to exclude any high-risk students. Candidates residing in a Red Zone area (which has more than 40 infected cases) or those with symptoms suspicious of COVID-19 infection are required to do undergo a COVID-19 test 48 hours prior to the exams^8^. These questionnaires had to be returned at least seven days prior to the exams. This was by no means an easy feat. With the exam 10 days away this left us 3 days to collect 140 replies. We also had to ensure that apart from those from Sabah, other candidates did not originate from an area of red zone.

Two examiners from Sarawak declined to attend as their university required 14-days quarantine upon re-entering Sarawak.

## Running the Exam

We were the first department to run a national level exam in our university during the COVID-19 pandemic period. Theory and OSCE exams went smoothly with untoward incidents. However, a few viva examiners expressed concerns with regards to social distancing measures and declined to attend the exam. One examiner cited advanced age. Some examiners also voiced doubt about the fact that our centre was not a Red Zone area. Consequently, the secretariat called all other examiners to ensure that there were no other concerns.

On the night of the second day of the exams, one area was declared a Red Zone. As stated above, candidates from Red Zone require a COVID-19 test 48 hours prior to the exams. With the exam well underway we were placed in a quandary. The Deans’ Council SOP did not mention what steps were to be taken if an area was declared a Red Zone in the midst of an exam. Certainly, performing COVID-19 test at this point was not an option. A decision was made to offer our two affected candidates an online viva session via Zoom. They declined stating preference to defer the viva section to the next exam. The candidates felt that an online session would not give them the opportunity to use writing medium to aid them when answering certain questions such as drawing a graph. Other stated reasons include unfamiliarity with Zoom, lack of proper facility (laptop with camera) and potential poor internet connection.

In retrospect, the Anatomy section would have been difficult to conduct via Zoom or online medium. Furthermore, we would have had no way of ensuring confidentiality during an online viva. Questions arise on how one could determine that students were indeed alone and received no assistance from another source. In addition to the two candidates, one examiner was also affected and was thus excluded from participating in the exam. Since then, further guidelines were implemented. From this point, daily checks on Red Zone is mandatory. During the remainder of the exam there were no other major issues. Candidates were continuously reminded not to congregate at the examination entrance and to come 30 minutes prior to the exams. Despite our meticulous planning, we discovered that our dining space was still inadequate requiring us to set up more tables in an adjacent area. We opted for a buffet layout and thus placed extra staff to serve food to the examiners.

## Recommendations

Based on our experience with running the OSCE Part I exam, we suggest the following recommendations for future exams during this pandemic:

1. Venue (Examination hall, wards, quarantine area, registration area) – double or triple the space of a normal setting
2. Questionnaires – Prepare a health risk assessment questionnaire for all candidates
3. Equipment – Thermometer scan, 3-ply surgical masks, face shields, aprons and gloves
4. Prepare QR code for scanning
5. Examiners –
  - Reserve examiners should be double the usual numbers
  - Prudent to obtain examiners without co-morbidities or of a younger age group
6. Avoid stations with standardised patients
7. Daily checks on declaration of Red Zone areas as well as University, Ministry of Health and government guidelines
8. Online and audio-visual facilities for possible online viva sessions
9. Candidates to reside near the examination venue to prevent inability to attend the examination if red zones are declared
10. Food and beverage – packed food recommended

## Conclusion

The COVID-19 pandemic is an unprecedented event in our lifetime. However, this does not mean that normal life should stop. Examinations are a normal process in producing qualified orthopaedic surgeons. Every effort should be made to prevent cancellation or postponement. During these uncertain times we must be able to respond quickly to dynamic circumstances and have alternative options available.
